# A Multidisciplinary Investigation to Determine the Structure and Source of Dimeric Impurities in AMG 517 Drug Substance

**DOI:** 10.1155/2009/768743

**Published:** 2008-12-17

**Authors:** Maria Victoria Silva Elipe, Zhixin Jessica Tan, Michael Ronk, Tracy Bostick

**Affiliations:** ^1^Department of Analytical Research and Development, Amgen Inc., Thousand Oaks, CA 91320, USA; ^2^Genentech Inc., One DNA Way, Mail Stop 432A, South San Francisco, CA 94080, USA; ^3^Chemical Process Research and Development Department, Amgen Inc., Thousand Oaks, CA 91320, USA

## Abstract

In the initial scale-up batches of the experimental drug substance AMG 517, a pair of unexpected impurities was observed by HPLC. Analysis of data from initial LC-MS experiments indicated the presence of two dimer-like molecules. One impurity had an additional sulfur atom incorporated into its structure relative to the other impurity. Isolation of the impurities was performed, and further structural elucidation experiments were conducted with high-resolution LC-MS and 2D NMR. The dimeric structures were confirmed, with one of the impurities having an unexpected C-S-C linkage. Based on the synthetic route of AMG 517, it was unlikely that these impurities were generated during the last two steps of the process. Stress studies on the enriched impurities were carried out to further confirm the existence of the C-S-C linkage in the benzothiazole portion of AMG 517. Further investigation revealed that these two dimeric impurities originated from existing impurities in the AMG 517 starting material, N-acetyl benzothiazole. The characterization of these two dimeric impurities allowed for better quality control of new batches of the N-acetyl benzothiazole starting material. As a result, subsequent batches of AMG 517 contained no reportable levels of these two impurities

## 1. Introduction

In the early stages of new drug development, understanding the impurity profiles of the drug substance is critical when interpreting the data from toxicology and clinical studies. There is a body of regulatory requirements with regard to identification and control of impurities. A commonly used framework used in the pharmaceutical industry is Q3A(R2), the International Conference on Harmonization (ICH) guidance for controlling impurities in new drug substance [1]. Although this guidance is intended only for products approaching application for final market registration, many companies consider similar elements when evaluating impurities in new chemical entities during the clinical phases of development.

Impurities in drug substances are classified into several categories in the ICH guideline Q3A(R2): organic impurities, inorganic impurities, and residual solvents. The organic impurities are of major concern for a new drug substance produced by chemical synthesis because the potential toxicity of most of these impurities is unknown. These impurities can originate from starting materials, by-products, intermediates, degradation products, reagents, ligands, and catalysts [1]. Knowledge of impurity structures can provide important insight into the chemical reactions responsible for forming these impurities as well as understanding potential degradation pathways [2]. Such information is essential in establishing critical control points in the drug substance synthetic process and eventually ensuring its overall quality and safety.

HPLC with UV detection is the most common analytical methodology used in the pharmaceutical industry to monitor organic impurities in new drug substances [2, 3]. These HPLC-UV methods are frequently used to track impurity profiles across various batches of drug substance which are often produced by different synthetic routes and at different scales. This is especially important in the earlier phases of clinical development when, due to resources and time constraints, the synthetic process is dynamic and not completely characterized, and the source/quality of starting materials has not been thoroughly evaluated [4]. When a new impurity is detected above a particular threshold (e.g., >0.10% according to ICH Q3A(R2) for commercial products), structural elucidation of that impurity is typically initiated. LC-MS systems are widely available these days and are routinely used in initial impurity identification efforts during early drug development phases [5]. The sensitivity of LC-MS allows for the analysis of the impurities without isolation, which is often time consuming. Coupled with knowledge of the sample's history (e.g., synthetic scheme, purification process, storage conditions, stress conditions, etc.), it is often possible to propose the chemical structure of the impurity solely based on LC-MS data [6, 7]. However, the LC-MS data alone may not provide sufficient information to derive a chemical structure. In such cases, NMR spectroscopy (1D and/or 2D) is often employed to gather further structural information for impurity identification [8, 9]. Although online LC-NMR has gained some popularity in recent years [10, 11], isolation or enrichment of impurity component for offline NMR studies is still one of the most common approaches [12, 13]. Frequently, publications detailing the identification of pharmaceutical impurities will focus on the application of a selected technique and will document the proposed formation reaction for the impurity. Rarely does the publication involve multiple analytical disciplines used to both identify the impurity and to trace back to its ultimate source through a complex synthetic scheme [14].

**Figure 1:**
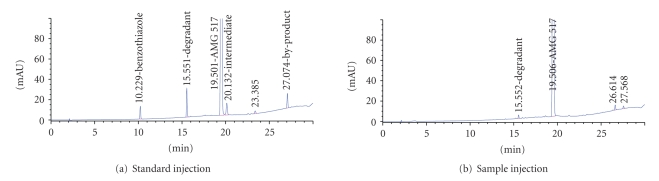
(a) HPLC-UV chromatogram of a standard mixture and (b) a representative AMG 517 sample containing the unknown impurities. Chromatographic conditions are in the experimental section and Table 1.

**Figure 2:**
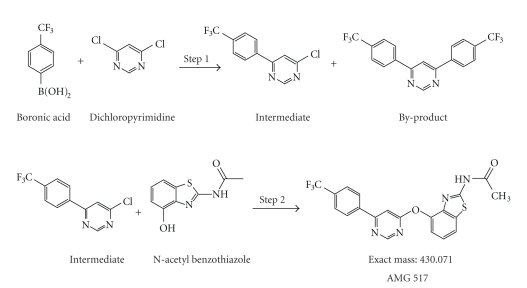
Synthetic pathway of AMG 517 during the early stages of clinical development.

Preparation for the first kilogram-scale production of one of Amgen's investigational anti-inflammatory drugs, AMG 517, provides a case in which a multidisciplinary investigation involving HPLC-UV, LC-MS, NMR, preparative HPLC, and forced degradation was required for unequivocal impurity identification. Two unexpected late eluting impurities were detected by an HPLC-UV method during release testing of this first scale-up batch of AMG 517 (see Figure 1). This first kilogram-scale batch of AMG 517 was manufactured with a process that was not well characterized (see Figure 2), using starting materials from outside vendors with which we had very little prior experience. Such situation is not uncommon in early clinical drug development. As the new batch was slated for use in first-in-human clinical trials, characterization of these impurities was required to enable process development which would lead to better process control. As a result of LC-MS and NMR analyses, the structures of these impurities were proposed as a simple dimer of AMG 517 and a thioether-linked dimer. A typical impurity investigation may end here with proposal of impurity structures. However, the formation of these impurities could not be explained by the synthesis scheme shown in Figure 2. A forced degradation study of the dimeric impurities provided a degree of certainty to the proposed structure for the thioether impurity. The desire to understand the origin of these impurities in the drug substance led to investigation of starting materials using HPLC-UV and LC-MS. Information compiled from these studies allowed us to work back through the synthetic scheme for AMG 517 to determine the source of the dimeric impurities. Knowing the origin of these impurities ultimately allowed for better quality control of the AMG 517 drug substance.

## 2. Experimental

### 2.1. Materials and Reagents

HPLC grade acetonitrile (ACN, Burdick and Jackson, Muskegon, Mich, USA), trifluoroacetic acid (TFA, J. T. Baker, Phillipsburg, NJ, and Pierce, Rockford, Ill, USA), and purified water from a Milli-Q unit (Millipore, Molsheim, France) were used in the preparation of various mobile phases and diluents in chromatographic analysis. Dimethyl-*d6* sulfoxide (DMSO-*d6*) “100%” (D, 99.96%), used for NMR analysis, was from Cambridge Isotope Laboratories (Andover, Mass, USA).

Samples of AMG 517 drug substance, N-(4-hydroxy- benzo[d]thiazol-2-yl)acetamide (N-acetyl benzothiazole), and the enriched impurity fraction were provided by the Chemical Process Research and Development Department of Amgen inc., (Thousand Oaks, Calif, USA).

### 2.2. HPLC

Analytical-scale chromatographic analyses were performed on an Agilent (Wilmington, Del, USA) 1100 series HPLC system. Mobile phase A was 0.1% TFA in water; mobile phase B was 0.1% TFA in ACN. A Phenomenex (Torrance, Calif, USA) Luna C18(2) HPLC column (5  *μ*m, 150 × 4.6 mm, at 30°C) was used for the separation and quantitation of the AMG 517 impurities. Two different gradients with different flow rates were employed for the separation of AMG 517 and N-acetyl benzothiazole (see Table 1). A UV detection wavelength of 254 nm and an injection volume of 30  *μ*L were used in the analysis of both compounds.

### 2.3. LC-MS

LC-MS experiments with accurate mass determination via high resolution mass spectrometry were performed using an Agilent 1100 HPLC (configured with a diode array UV detector) interfaced with a Waters (Milford, Mass, USA) Micromass Q-Tof Ultima API quadrupole time-of-flight mass spectrometer. The mass spectrometer was configured with a lockspray electrospray ionization (ESI) source to allow for the introduction of an internal mass calibration solution, which provides for a 5 ppm mass error specification when used in conjunction with tune settings producing ~20 000 mass resolution on the instrument.

LC-MS analyses of the enriched impurities, and of their hydrolysates, were accomplished using a Phenomenex Luna C18(2) HPLC column (3  *μ*, 100 Å, 2.0 × 150 mm) and mobile phase consisting of 0.1% aqueous TFA (mobile phase A) and 0.1% TFA in ACN (mobile phase B). A flow rate of 0.2 mL/minute was used, and a column temperature of 30°C was maintained throughout each HPLC run. Gradient conditions listed in Table 1 for the HPLC-UV analysis of AMG 517 were also used for the LC-MS analysis of AMG 517 and its impurities.

LC-MS analysis of the AMG 517 starting material, N-acetyl benzothiazole, was accomplished using a Phenomenex Luna C18(2) HPLC column (3  *μ*, 100 Å, 4.6 × 150 mm). The same mobile phase system described above was used at a flow rate of 1.0 mL/minute. Column temperature was also maintained at 30°C. The gradient conditions used for the LC-MS analysis of N-acetyl benzothiazole are listed in Table 1.

### 2.4. NMR

Spectra were acquired at 25°C and 27°C on Bruker DPX 400 and Bruker AVANCE 600 NMR instruments (Bruker BioSpin Corporation, Billerica, Mass, USA) equipped with 5 mm and 2.5 mm multinuclear inverse z-gradient probes, respectively. ^1^H NMR experiments were carried out at 400.13 and 600.13 MHz, respectively, and ^13^C NMR experiments were carried out at 100.61 and 150.90 MHz, respectively. The data processing was performed on the spectrometers. Chemical shifts are reported in the *δ* scale (ppm) by assigning the residual solvent peak at 2.50 and 39.51 ppm to DMSO for ^1^H and ^13^C, respectively. The 1D ^1^H and ^13^C NMR spectra were determined using a 30° flip angle with 1 second and 2 seconds equilibrium delays, respectively. The 90° pulses used were 7.7 and 4.5 microseconds for ^1^H, and 22.0 and 12.50 microseconds for ^13^C in experiments carried out on the 400 and 600 MHz spectrometers, respectively. The ^1^H, ^1^H-2D correlation spectroscopy (COSY) spectra were acquired into 2K data points in the *f*2 dimension with 128 increments in the *f*1 dimension, using a spectral width of 4789.3 Hz on the 400 MHz spectrometer and 7788.2 Hz on the 600 MHz instrument. The nuclear Overhauser effect spectroscopy (NOESY) experiments were determined with an 800 milliseconds mixing time, and with the same spectral width for *f*2 dimensions as COSY experiments, but with 256 increments in *f*1 dimension. The delays between successive pulses were 1.5 and 2 seconds for 2D COSY and NOESY, respectively. Both the ^1^H, ^13^C-2D heteronuclear single-quantum correlation (HSQC) and ^1^H, ^13^C-2D heteronuclear multiple bond correlation (HMBC) spectra were determined using gradient pulses for coherence selection. The ^1^H, ^13^C-2D heteronuclear multiple-quantum correlation (HMQC) and the HSQC spectra were determined with decoupling during acquisition. The 2D HMQC and 2D HMBC experimental data were acquired on the 400 MHz spectrometer with spectral widths of 4789.3 Hz for ^1^H and 20123.9 Hz for ^13^C, into 1K data points in the *f*2 dimension with 128 increments in the *f*1 dimension. The 2D HSQC and 2D HMBC experimental data carried out on the 600 MHz spectrometer were acquired with spectral widths of 6009.6 and 7788.2 Hz for ^1^H for HSQC and HMBC, respectively, and 27162.5 Hz for ^13^C dimension. The data were acquired into 1K and 4K data points in the *f*2 dimension for HSQC and HMBC, respectively, and with 256 and 128 increments in the *f*1 dimension for HSQC and HMBC, respectively. Delays corresponding to one bond ^13^C–^1^H coupling (ca. 145 Hz) for the low-pass filter and to two-to-three bond ^13^C–^1^H long range coupling (7.7 Hz) were used for the HMBC experiments. All 2D NMR data were processed using sine and qsine weighting window functions with some line broadening.

## 3. Results and Discussion

### 3.1. Impurity Profiles in Kilogram-Scale Batches of AMG 517 Drug Substance

A stability indicating HPLC-UV method was developed to separate and quantify AMG 517 along with its potential impurities and possible degradants. Figure 1(a) represents a typical separation of a standard mixture of AMG 517 in the presence of its known impurities and degradants. This method was used to analyze the first six drug substance batches of AMG 517 during release and stability testing of these lots. A pair of unexpected late-eluting unknown impurities was observed in all six batches of AMG 517. The area percent levels of impurity Unknown 1 ranged from 0.15% to 0.44%, while Unknown 2 ranged from 0.06% to 0.21%. A representative chromatogram of an AMG 517 drug substance lot containing these impurities is shown in Figure 1(b). These two impurities were not detected at a reportable level in the previous small-scale batches of AMG 517. Since these two unknown impurities eluted near the retention time of the by-product in step 1 of the AMG 517 synthetic reaction, it was concluded that these new impurities were highly hydrophobic and may have structural features similar to the by-product (see Figure 2).

Preliminary low-resolution LC-MS analysis on the drug substance provided molecular mass and tandem mass spectrometry (MS/MS) fragment ion information for these two impurities (data not shown). The observed mass for the protonated Unknown 1 and Unknown 2 was 859 Da and 891 Da, respectively. Since the exact mass for AMG 517 is 430.0711 Da, an observed mass of 859 Da for Unknown 1 suggested that it could be some sort of dimeric structure related to AMG 517. MS/MS data also suggested dimeric structures for both impurities. Fragment ions that corresponded to the neutral loss of multiple acetyl and hydrofluoric functional groups were observed in MS/MS experiments performed on the protonated ions of both Unknown 1 and Unknown 2. The mass difference between the two unknowns was 32 Da, which could be attributed to either one additional sulfur atom or two additional oxygen atoms in Unknown 2 relative to Unknown 1. However, the preliminary LC-MS analysis alone could not conclusively identify the structures of these impurities due to the possible existence of multiple isomeric structures consistent with the mass data. To aid in the structural elucidation efforts, an enriched fraction of these two impurities was isolated via preparative-scale HPLC. The isolated fraction contained about 35% of Unknown 1 and 62% of Unknown 2 based on UV detection at 254 nm. LC-MS and NMR experiments were performed to characterize this enriched fraction.

### 3.2. Accurate Mass Determination for Unknowns 1 and 2 in the Enriched Fraction

An accurate mass of 859.1342 Da was determined for Unknown 1 in the enriched fraction. Elemental composition analysis was performed for this protonated mass. Instrument performance, the synthetic pathway for AMG 517, and information gained from the preliminary LC-MS analysis of the impurities were taken into account in setting parameters for this analysis. Based upon the performance of the mass spectrometer, the error between the observed and calculated masses was limited to 5 ppm or less. MS/MS analysis indicated the presence of two trifluoromethyl groups, so the number of atoms of F required was set to six. MS/MS analysis also indicated the presence of two acetyl groups, so the minimum number of atoms of O required was set to two. Consideration of the synthetic pathway for AMG 517 suggested that a molecule containing less than four atoms of N was unlikely. All elemental composition analyses performed as part of this investigation utilized a similar strategy to logically identify the most likely elemental formula for an observed mass.

The elemental composition analysis for the observed mass of Unknown 1 determined that the elemental formula C_40_H_24_N_8_O_4_F_6_S_2_ was the best fit for the impurity. This elemental composition was consistent with a dimer of AMG 517 minus two hydrogens (elemental formula C_20_H_13_N_4_O_2_F_3_S). The mass error between the observed mass for Unknown 1 and the calculated mass for a dimer of AMG 517 was 0.3 ppm.

An accurate mass of 891.1058 Da was determined for Unknown 2 in the enriched fraction. Elemental composition analysis using this protonated mass determined that the elemental formula C_40_H_24_N_8_O_4_F_6_S_3_ was the best fit for the impurity. This elemental composition was consistent with a dimer of AMG 517 with the addition of a sulfur atom [dimer+S]. The mass error between the observed mass for Unknown 2 and the calculated mass for [dimer+S] was 0.8 ppm.

Another possible elemental formula for Unknown 2 is C_40_H_24_N_8_O_6_F_6_S_2_ which corresponded to an AMG 517 dimer with two additional oxygen atoms [dimer+2O]. The mass error between the observed mass for Unknown 2 and the calculated mass for the [dimer+2O] was 20.7 ppm. Based on the accurate mass data, it was concluded that [dimer+S] was a more likely structure for Unknown 2.

The MS data was consistent with dimeric structures for both Unknown 1 and Unknown 2 but provided no definitive structural linkage information. The structure of AMG 517 itself and the synthetic scheme shown in Figure 2 did not provide any obvious possible point of linkage. Therefore, NMR analyses were performed on the enriched fraction to help elucidating the structures of these impurities.

### 3.3. NMR

AMG 517 and its enriched impurity fraction containing Unknowns 1 and 2 were first analyzed by ^1^H and ^13^C NMR to further investigate the connectivity. Proton assignments were made based on chemical shifts, proton-proton coupling constants, and COSY and NOESY spectra (see Tables 2 and 3). Carbon assignments were based on chemical shifts, carbon-fluorine coupling constants, and HMQC, HSQC, and HMBC spectra (see Tables 2 and 3). All assignments referring to the structures of AMG 517 and impurities are depicted in these two tables.

NMR analysis was also conducted on AMG 517 for comparison (see Table 2). The ^1^H NMR spectrum showed the presence of all the protons of the molecule including the exchangeable NH proton. The ^1^H NMR spectrum showed the presence of three aromatic systems, an AA'BB' spin system (*δ* 7.92 and 8.44 ppm) for a *p*-disubstituted benzene ring, two singlets (*δ* 7.97 and 8.79 ppm) for another aromatic ring, and an ABX spin system (*δ* 7.35, 7.39, and 7.93 ppm) for a 1,2,3-trisubstituted benzene ring. The downfield chemical shift of the singlet at 8.79 ppm together with the singlet at 7.97 ppm suggested a 4,6-disubstituted pyrimidine as one of the aromatic rings in the molecule. The ^13^C NMR spectrum showed the presence of all the carbons of the molecule. Three of these carbons were coupled to ^19^F; C-13 as a quartet through one C–F bond (*δ* 124.0, ^1^J[^13^C, ^19^F] = 272.2 Hz), C-1 as a quartet through one C–C and one C–F bonds (*δ* 130.9, ^2^J[^13^C, ^19^F] = 31.9 Hz), and C-2, 6 as a quartet through two C–C and one C–F bonds (*δ* 125.9, ^3^J[^13^C, ^19^F] = 3.7 Hz) (see Table 2).

**Figure 3:**
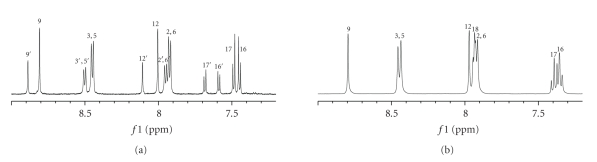
Aromatic region of the ^1^H NMR spectra of the enriched impurity fraction ((a) 600 MHz) and AMG 517 ((b) 400 MHz) in DMSO-*d6.* In (a), numbers designated as prime (e.g., 3′) represent signals of Unknown 1, with all others representing signals of Unknown 2.

**Figure 4:**
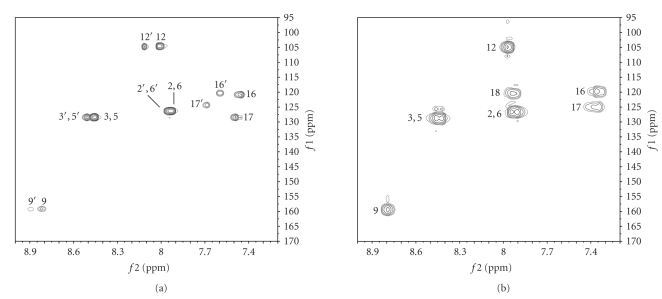
Aromatic region of the ^1^H, ^13^C-2D HSQC spectrum of the enriched impurity fraction ((a) 600 MHz) and the ^1^H, ^13^C-2D HMQC spectrum of AMG 517 ((b) 400 MHz) in DMSO-*d6*. In (a), numbers designated as prime (e.g., 3′) represent signals of Unknown 1, with all others representing signals of Unknown 2.

The ^1^H NMR spectrum of the enriched fraction containing the impurities indicated that the sample was a mixture of two components structurally related to AMG 517, present at a ratio of 1:1.94 based on the areas of their related aromatic signals. Based on the HPLC-UV data from the enriched fraction, the major component present corresponded to Unknown 2, and the minor component to Unknown 1. The ^1^H NMR spectrum of the impurities contained signals corresponding to the same substitution patterns observed for AMG 517 (see Figure 3). ^1^H NMR and ^1^H, ^1^H-2D NOESY spectra indicated the presence of a *p*-disubstituted benzene ring, a 4,6-disubstituted pyrimidine, a 2,4,7-trisubstituted benzothiazole ring, and an N-acetyl group. The only difference between AMG 517 and these two related compounds is the substitution pattern of the benzothiazole. The ^1^H NMR spectrum showed more distinct chemical shift differences for the protons H-16 and H-17 from these two AMG 517-related compounds (see Figure 3 and Table 4). The signals from Unknown 1 were shifted downfield compared to Unknown 2. The elemental molecular formulae for Unknowns 1 and 2 were indicative of dimer structures. Only one set of resonances was observed for each of the two unknowns. This indicated that the unknowns were symmetrical dimers. The monomers were connected through carbon C-18 based on the presence of an AB system, their chemical shifts, and the coupling constants for the benzothiazole ring. The ^1^H, ^13^C-2D HSQC spectrum supported the ^1^H NMR data showing only two aromatic C–H (C-16 and 17) on the benzothiazole ring of the impurities. The absence of a C–H signal for C-18, as was observed in AMG 517, was noted in the NMR spectra in both of the unknowns (see Figure 4). ^13^C NMR, ^1^H, ^13^C-2D HSQC, and ^1^H, ^13^C-2D HMBC spectra of the impurities showed more distinct chemical shift differences for the carbons C-16, C-17, C-18, and C-19. This indicated that the difference between these two impurities was in the linkage through C-18, either directly or through a heteroatom (see Table 4). The possibility of having a sulfur atom connecting the two AMG 517 monomers for Unknown 2 was considered very plausible based on the MS data and the chemical shift data (see Table 4).

### 3.4. LC-MS Analysis of the Hydrolysate of the Enriched Impurities

The MS data for the two impurities strongly supported a thioether-linked dimer of AMG 517 as the structure for Unknown 2. The ^1^H and ^13^C NMR data provided indirect evidence of such thioether linkage but could not afford direct measurement of the heteroatom. However, the formation of this impurity in the synthesis of AMG 517 (see Figure 2) did not seem as plausible as the oxidation of a heteroatom from a reaction mechanistic standpoint. There was a significant difference between the calculated mass values for the two potential structures for Unknown 2, however, the relatively high mass of the impurity resulted in a large number of potential elemental formulae. To simplify the elemental composition analysis, a chemical degradation experiment was performed. The enriched fraction was treated with 0.5 equivalent of aqueous HCl in DMSO-*d6* and heated overnight at 70°C. This experiment furnished low-molecular-weight fragments of the impurity that could not be generated via MS/MS. These low-mass fragments resulted in a small number of potential elemental formulae for each observed mass.

**Figure 5:**
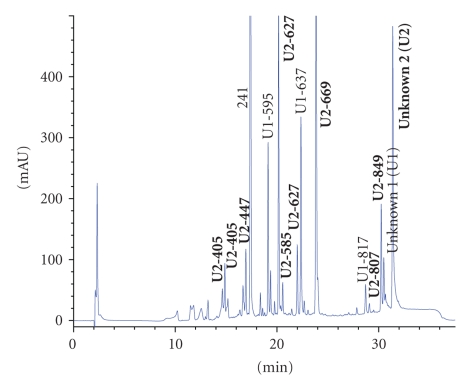
UV chromatogram from the LC-MS analysis of the acid hydrolyzed impurities. Labeled peaks correspond to hydrolysis fragments of Unknown 1 (U1) and Unknown 2 (U2).

**Figure 6:**
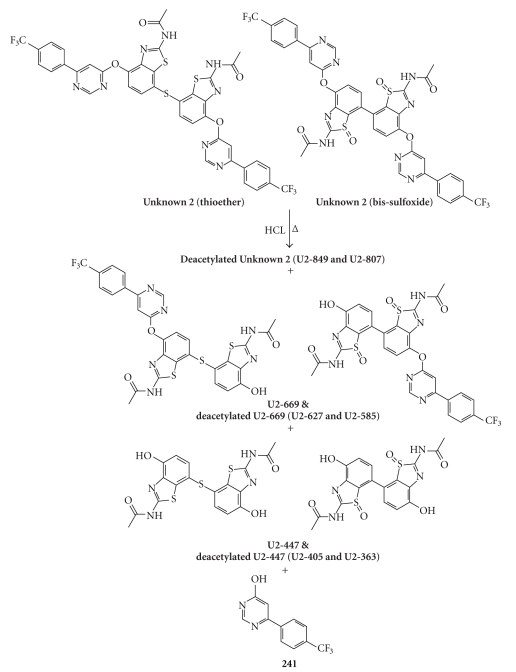
Potential fragments produced by acid hydrolysis of Unknown 2. Structures on the left represent fragments expected to be generated from the thioether, those on the right from the bis-sulfoxide. Fragment 241 would be common to both structures.

Multiple hydrolysis fragments were observed in LC-MS after forced degradation of the enriched fraction with hydrochloric acid (see Figure 5). Accurate mass data collected in the LC-MS analysis of the acid treated enriched impurity fraction was used to identify peaks corresponding to the expected hydrolysis fragments (see Figure 5). The scheme in Figure 6 shows the expected acid hydrolysis fragments from Unknown 2, with Unknown 2 and its fragments presented using both the thioether and bis-sulfoxide structures being considered for the impurity. A number of deacetylation products were also observed. This LC-MS analysis demonstrated that all of the expected fragments for Unknown 2 (and some for Unknown 1) were formed during the forced degradation.

Table 5 shows the accurate mass assignments for the acid hydrolysis products of Unknown 2 as well as the calculated exact mass for each product that was expected to arise from both the proposed thioether and bis-sulfoxide structures. The mass error (observed mass versus calculated mass of the hydrolysis fragments) is shown for each proposed structure for Unknown 2. The mass error range for hydrolysis products arising from the thioether structure was 0 to 3.5 ppm; the mass error range for the corresponding bis-sulfoxide was 17.5 to 40.7 ppm. Thus, the accurate mass data collected for the acid hydrolysis fragments allowed for elimination of the bis-sulfoxide as a potential structure for Unknown 2.

These mass error results strongly supported an elemental formula of C_40_H_24_F_6_N_8_O_4_S_3_ (thioether) for Unknown 2, and essentially ruled out an elemental formula of C_40_H_24_F_6_N_8_O_6_S_2_ (bis-sulfoxide) for the impurity.

### 3.5. LC-MS Analysis of N-Acetyl Benzothiazole Starting Material

Although the MS and NMR data provided great confidence in the proposed dimeric structures for these two late-eluting impurities, the chemical reactions described in Figure 2 were not likely to generate such impurities. Since the dimeric linkages are in the benzothiazole portion of AMG 517, it was possible that these two impurities were originated from existing impurities in the AMG 517 starting material, N-acetyl benzothiazole, which was prepared via multistep synthesis from 2-methoxybenzenamine by a contract manufacturer. To determine if N-acetyl benzothiazole was a potential source for generating Unknowns 1 and 2, additional experiments were performed to evaluate the impurity profiles of N-acetyl benzothiazole.

**Figure 7:**
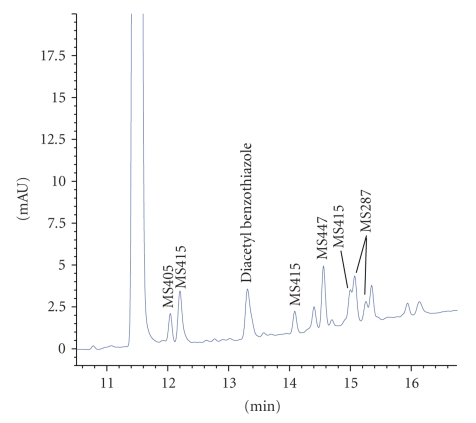
Expanded view of the UV chromatogram from the LC-MS analysis of N-acetyl benzothiazole lot A (ca. 0.1 mg/mL in 10% ACN, 3  *μ*g injected on column).

A different HPLC method was developed for the analysis of N-acetyl benzothiazole (see Table 1). Although the supplier's Certificate of Analysis indicated an HPLC purity of >99% area for various batches of N-acetyl benzothiazole, retrospective analysis by Amgen's HPLC method resulted in purities ranging from 96.2 to 98.2% area. LC-MS analysis was performed on lot A, the starting material used in the production of the six kilogram-scale AMG 517 batches. Analysis of this lot indicated that there were multiple impurities, some of which had the potential to generate Unknowns 1 and 2 (see Figure 7). These impurities were designated by their nominal mass values as determined by the LC-MS analysis (e.g., MS447 corresponds to a compound with an observed mass of 447 Da). Table 6 provides a summary of the proposed structures for the observed impurities of N-acetyl benzothiazole.

**Figure 8:**
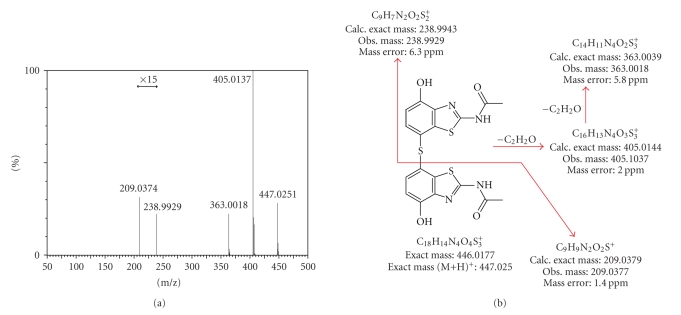
(a) MS/MS analysis (with accurate mass determination) of the protonated ion of MS447. (b) Schematic of the MS/MS fragmentation interpretation of MS447.

**Figure 9:**
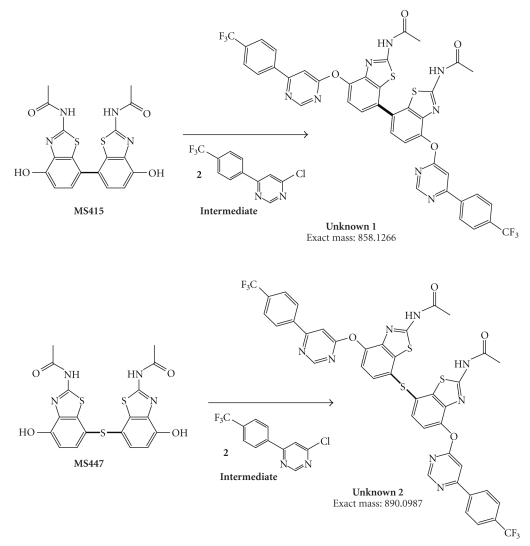
Proposed structures and formation pathway for the two unknown impurities in AMG 517.

An accurate mass of 447.0247 Da was determined for the protonated ion of impurity MS447 in N-acetyl benzothiazole. Elemental composition analysis using the observed mass determined that the elemental formula C_18_H_14_N_4_O_4_S_3_ was the best fit for this impurity. This elemental formula, along with MS/MS analysis of MS447 (see Figure 8), supported a thioether linked dimer of benzothiazole as the structure for MS447 (see Table 6). This symmetrical thioether compound could participate in the same reaction as AMG 517 step 2 to generate Unknown 2 (see Figure 9).

An accurate mass of 405.0144 Da was determined for the protonated ion of impurity MS405. Elemental composition analysis using the observed mass determined that the elemental formula C_16_H_12_N_4_O_3_S_3_ was the best fit for the impurity. MS405 was proposed to be the mono-deacetylated form of MS447.

Accurate mass determination for each of the three peaks designated MS415 led to the assignment of exact mass values that were in close agreement with each other (415.0522 Da, 415.0540 Da, and 415.0536 Da, in order of elution), and elemental composition analysis using these observed mass values points to the same elemental composition (C_18_H_14_N_4_O_4_S_2_) as the most likely formula for each. These three MS415 impurities in N-acetyl benzothiazole could be positional isomers to each other. One of these isomers, a symmetrical ortho dimer (see Table 6), was a plausible precursor for the proposed structure of Unknown 1 (see Figure 9).

Accurate mass determination for each of the two peaks designated MS287 led to the assignment of exact mass values that are in close agreement with each other (286.9486 Da and 286.9475 Da, in order of elution), and elemental composition analysis using these observed mass values points to the same elemental composition (C_9_H_7_N_2_O_2_SBr) as the most likely formula for each. Structures consistent with these elemental formulae are shown in Table 6. The presence of these molecules in the benzothiazole synthetic process could lead to the formation of impurities MS415 and MS447.

## 4. Conclusion

An extensive investigation successfully utilized multiple analytical disciplines to elucidate structures for two complex impurities in AMG 517 drug substance and to trace the source of the impurities to a starting material used in the manufacture of AMG 517.

The structures of two unknown impurities in AMG 517 drug substance were identified through extensive HPLC, LC-MS, high resolution MS, MS/MS, and 1D and 2D NMR studies. The existence of an unexpected C-S-C linkage in one of the impurities was confirmed. Further investigation revealed that these impurities originated from existing impurities in the N-acetyl benzothiazole starting material used in AMG 517 synthesis. This information was shared with the supplier of this starting material, and the process for N-acetyl benzothiazole preparation was re-evaluated. Better synthetic process controls and tighter specifications were established resulting in higher quality N-acetyl benzothiazole batches. These two dimeric impurities were not observed in subsequent larger-scale AMG 517 production runs.

## Figures and Tables

**Table 1:** Gradient conditions used for the HPLC-UV and LC-MS analyses of AMG 517 and N-acetyl benzothiazole.

Compound	AMG 517		N-acetyl Benzothiazole			
	(HPLC-UV and LC-MS)		(HPLC-UV)		(LC-MS)	
Gradient program	Time (min)	%B	Time (min)	%B	Time (min)	%B
	0	5	0	5	0	5
	15	65	10	30	10	30
	20	70	15	50	15	50
	27	98	20	75	20	75
	30	98			25	95

Flow rate	1.0 mL/min		1.5 mL/min		1.0 mL/min	

Sample diluent	50% ACN/50% water		10% ACN/90% water			

**Table 2:** ^1^H and ^13^C chemical shifts (*δ*/ppm) of AMG 517 standard in DMSO-*d6* (400 MHz).

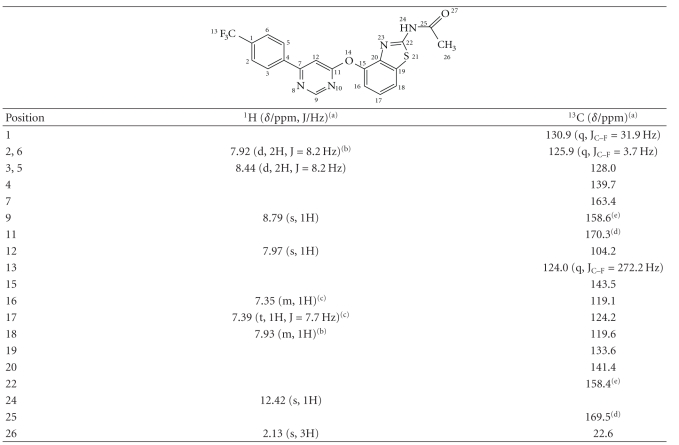

^(a)^Signal splitting patterns: s = singlet, d = doublet, t = triplet, q = quartet, m = multiplet; ^(b), (c)^overlapping; ^(d), (e)^interchangeable assignment.

**Table 3:** ^1^H and ^13^C chemical shifts (*δ*/ppm) of the enriched impurity fraction in DMSO-*d6*.

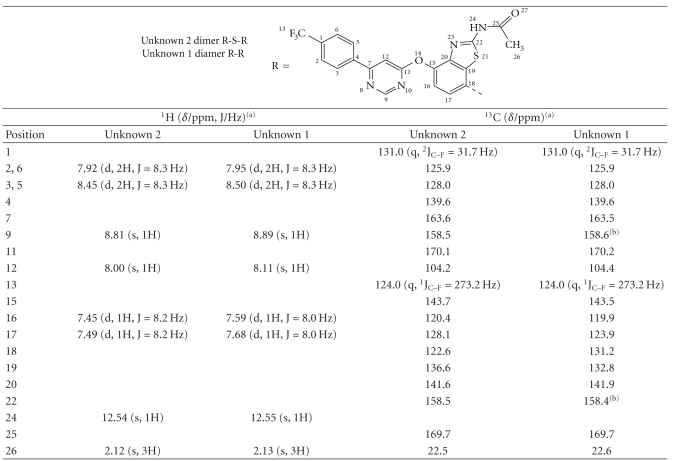

^(a)^Signal splitting pattern: s = singlet, d = doublet, q = quartet; ^(b)^interchangeable assignments.

**Table 4:** Partial ^1^H and ^13^C chemical shifts (**δ** /ppm) of the benzothiazole ring for AMG 517 and the enriched impurity fraction in DMSO-*d6*.

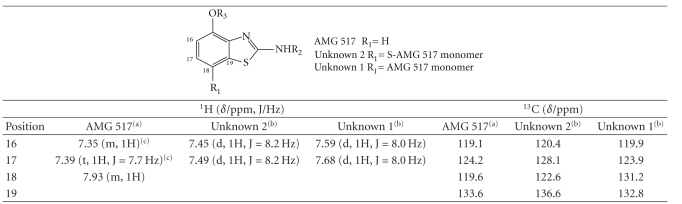

^(a)^Data from 400 MHz NMR instrument; ^(b)^data from 600 MHz instrument; ^(c)^overlapping signals.

**Table 5:** Mass error analysis of the observed accurate mass for fragments generated by the acid hydrolysis of the enriched impurity fraction. The analysis is conducted for both the thioether and bis-sulfoxide structures proposed for Unknown 2. (ND: not detected; N/A: not applicable.)

	Obs. mass (M + H)^+^ (Da)	Calc. mass thioether (M + H)^+^ (Da)	Calc. mass bis-sulfoxide (M + H)^+^ (Da)	Mass error thioether (ppm)	Mass error bis- sulfoxide (ppm)
Unknown 2	891.1081	891.1060	891.1237	2.4	17.5
Mono-deacetyl	849.0967	849.0954	849.1132	1.5	19.4
Bis-deacetyl	807.0874	807.0848	807.1026	3.2	18.8
U2-669	669.0674	669.0655	669.0832	2.8	23.6
Mono-deacetyl	627.0552	627.0549	627.0727	0.5	27.9
	627.0558			1.4	27.0
Bis-deacetyl	585.0458	585.0443	585.0621	2.6	27.9
U2-447	447.0250	447.0250	447.0428	0.0	39.8
Mono-deacetyl	405.0158	405.0144	405.0322	3.5	40.5
	405.0157			3.2	40.7
Bis-deacetyl	ND	363.0039	363.0216	N/A	N/A
241	241.0579	241.0583	241.0583	1.7	1.7

**Table 6:**
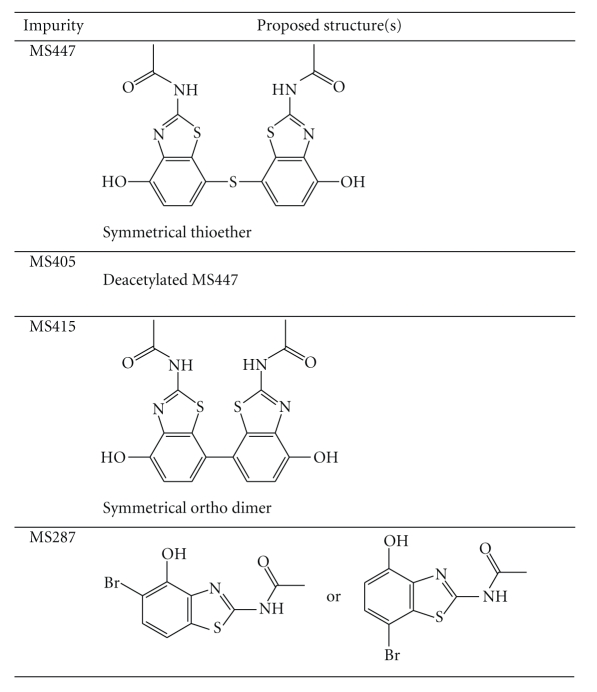
Summary of proposed structures of impurities observed in the LC-MS analysis of N-acetyl benzothiazole.
